# Effect of Al_2_O_3_ Dot Patterning on CZTSSe Solar Cell Characteristics

**DOI:** 10.3390/nano10091874

**Published:** 2020-09-18

**Authors:** Se-Yun Kim, Sanghun Hong, Seung-Hyun Kim, Dae-Ho Son, Young-Ill Kim, Sammi Kim, Young-Woo Heo, Jin-Kyu Kang, Dae-Hwan Kim

**Affiliations:** 1 Research Center for Thin Film Solar Cells, Daegu-Gyeongbuk Institute of Science and Technology (DGIST), Daegu 42988, Korea; kimseyun@kyungnam.ac.kr (S.-Y.K.); seunghyun@dgist.ac.kr (S.-H.K.); dhson@dgist.ac.kr (D.-H.S.); lynx012@dgist.ac.kr (Y.-I.K.); smkim@dgist.ac.kr (S.K.); 2 Department of Nano Materials Science and Engineering, Kyungnam University, Gyeongsangnam-do 51767, Korea; 3 School of Materials Science and Engineering, Kyungpook National University, Daegu 41566, Korea; shhong@dgist.ac.kr (S.H.); ywheo@knu.ac.kr (Y.-W.H.); 4 Division of Energy Technology, Daegu-Gyeongbuk Institute of Science and Technology (DGIST), Daegu 42988, Korea

**Keywords:** CZTSSe, metal precursor, two-step process, back-contact passivation, void arrangement

## Abstract

In this study, a 5-nm thick Al_2_O_3_ layer was patterned onto the Mo electrode in the form of a dot to produce a local rear contact, which looked at the effects of this contact structure on Cu_2_ZnSn(S_1-x_Se_x_)_4_ (CZTSSe) growth and solar cell devices. Mo was partially exposed through open holes having a square dot shape, and the closed-ratios of Al_2_O_3_ passivated areas were 56%, 75%, and 84%. The process of synthesizing CZTSSe is the same as that of the previous process showing 12.62% efficiency. When the 5-nm-Al_2_O_3_ dot patterning was applied to the Mo surface, we observed that the MoSSe formation was well suppressed under the area coated of 5-nm-Al_2_O_3_ film. The self-alignment phenomenon was observed in the back-contact area. CZTSSe was easily formed in the Mo-exposed area, while voids were formed near the Al_2_O_3_-coated area. The efficiency of the CZTSSe solar cell decreased when the Al_2_O_3_ passivated area increased. The exposure area and pitch of Mo, the collecting path of the hole, and the supplying path of Na seemed to be related to efficiency. Thus, it was suggested that the optimization of the Mo-exposed pattern and the additional Na supply are necessary to develop the optimum self-aligned CZTSSe light absorber.

## 1. Introduction

The efficiencies of CdTe and CIGS solar cells are reported to be close to those of Si solar cells, at 22.1% and 23.4%, respectively [1]. Unlike Si thin-film solar cells, CdTe and CIGS materials have a significant light absorption coefficient. They can thus absorb light sufficiently in thin films for solar cells [2]. Therefore, CdTe and CIGS thin-film solar cells are the most likely candidates to be applied to building-integrated photovoltaics (BIPVs) and vehicle-integrated photovoltaics (VIPVs) as flexible solar cells. However, because CdTe and CIGS materials have problems of Cd toxicity and supply and demand instability due to the scarcity of In and Ga, it is necessary to develop solar cells using abundant, non-toxic materials with no stability issues. As one of the emerging solar cell materials, halide perovskite materials have shown excellent characteristics [1,2]. Even so, there continue to be doubts about halide perovskite materials’ potential for commercialization due to photo, humidity, and thermal stability issues [3,4,5,6,7]. Another promising material is CZTSSe, which is a non-toxic and abundant material with high absorption coefficient [1,8,9]. Unfortunately, unlike CIGS, CZTSSe has not overcome the 12.6% efficiency barrier achieved in 2013 by the hydrazine solution process [10]. The origin of the barrier was estimated as follows: 1) Cu + Zn disordered kesterite has the lowest formation energy, causing additional problems such as energy bandgap fluctuation and a large amount of defect formation [11,12,13]. 2) The highly stable ZnS(e) phase [14,15], which always participates in the reaction pathway [16], makes it difficult to form a CZTSSe layer with a uniform composition. SnZn, which is a killing detect, is well-formed at conditions of Zn-poor kesterite [17,18,19]. 3) The Sn multivalent state is easily formed at a low partial pressure of chalcogenide [18,20]. 4) SnS loss occurs during the cooling process by CZTSSe decomposition at high temperatures [21,22]. 

Recently, it has been reported that the solar cell characteristics of CZTSSe synthesized using metal precursors exhibit high efficiency [15,23,24,25,26,27,28]. Our group has tied the record of the worlds’ highest efficiency using H_2_S gas and Se pellets. [23]. Since our process was performed at relatively high Se pressures and low temperatures, we believe that the impact on problems 3) and 4) has been reduced. According to our CZTSSe synthesis process, which shows efficiency of 12.62%, the CZTSSe absorption layer has the following unique microstructure. A CZTSSe double layer consisting of a dense upper CZTSSe layer and bottom CZTSSe layer that is partially composed of voids. [15]. Moreover, between the upper CZTSSe and bottom CZTSSe, the ZnSSe secondary phase remains as an unreacted residue [15]. Cu- and Cu-Sn-SSe secondary phases are distributed in the Mo back contact region [15]. According to the results of the formation mechanism investigation of the Cu- and Cu-Sn-SSe phases in the Mo-back contact region, the secondary phase formation can be suppressed by controlling the wetting characteristic of the Mo-back contact side [29]. When the Al_2_O_3_ coated Mo/SLG substrate is used, the formation of the secondary phase distributed at the Mo back contact region is completely suppressed [29]. However, void formation at the bottom CZTSSe layer of the CZTSSe double layer is inevitable when using Sn/Cu/Zn metal precursors [15,23,24,25,26]. Further experiments have shown that using a 130-nm sputtered Al_2_O_3_ line patterned Mo / SLG substrate, voids present in the bottom CZTSSe layer could be arranged in the Al_2_O_3_ coated area while the bottom CZTSSe was arranged on the Mo exposed area [26]. In previous studies, one type of line pattern was applied and MoSSe formation control was not performed [26]. Nevertheless, it was meaningful for the first time to show the possibility of arranging the irregular distribution of the bottom CZTSSe as desired.

In this study, a 5-nm-Al_2_O_3_ layer was deposited on the Mo electrode to suppress the MoSSe formation perfectively. By changing the distribution of square dots of the same size, the open ratio was changed to 44%, 25%, and 16%, and the passivation ratios were 56, 75, and 85%. Sizing of the Mo-exposed dot and the distance between each dot was designed based on the ratio of void and size of the bottom CZTSSe in the previous study. Mo-exposed square dots were formed by the photolithography process to control the distribution of the bottom CZTSSe. The effects of the passivated emitter and rear cell (PERC) and defect passivation at back contact side may be caused by Al_2_O_3_ intermediate layer, which may increase the cell efficiency [30,31,32]. In the case of solar cell characteristics, as the Al_2_O_3_ coated area increased, the efficiency decreased. The origin of reduced cell efficiency depends on the 5-nm-Al_2_O_3_ dot patterning distribution, which was speculated to be due to the insufficient diffusion of Na from SLG and non-optimized patterning for collecting the photo-generated carrier.

## 2. Materials and Methods

A BOE solution etched the Mo-deposited SLG substrate, and a 5-nm-thickness Al_2_O_3_ layer was deposited using ALD. In order to form the dot pattern, the general photolithography process was conducted using a photo-mask. The Sn (275 nm)/Cu (160 nm)/Zn (188 nm)/Mo stacked metal precursors for the CZTSSe absorber layer were deposited using 99.99% pure Sn, Cu, and Zn sputtering targets. A quartz box was used for the sulfo-selenization process with a SiC-coated graphite holder as the sample holder. Several Se shots (0.2 g, Sigma-Aldrich, Inc. St. Louis, United States) were placed on the quartz boat’s bottom, and the graphite holder was placed in the quartz box. The vaporized Se source flowed into the sample through the holes that were designed on four sides of the SiC-coated graphite holder. H_2_S (250 sccm) and Ar (2000 sccm) gas were supplied until the chamber pressure reached 700 Torr. The sample was heated at 300 °C for 15 min and then heated to 480 °C for 10 min.

A 50-nm-thick CdS buffer layer was deposited by chemical bath deposition. RF-sputtering deposited the intrinsic 50-nm-thick ZnO layer and 300-nm-thick Al-doped ZnO layer. Finally, a 20-nm-thick Ni and 2-µm-thick Al grid were deposited via e-beam evaporation.

The microstructure of the CZTSSe layer was characterized by FESEM (Hitachi, SU8020, Tokyo, Japan). The depth profile of each component of CZTSSe was analyzed by TOF-SIMS (ION TOF. TOF-SIMS 5-100, Heisenbergstraße, Germany). The solar cells were characterized according to the 1.5 AM illuminated J–V characteristics (94022A solar simulator Newport Co., Keithley 4200 semiconductor characterization system, Berkshire, United Kingdom) in our laboratory.

## 3. Results

**Figure 1:**
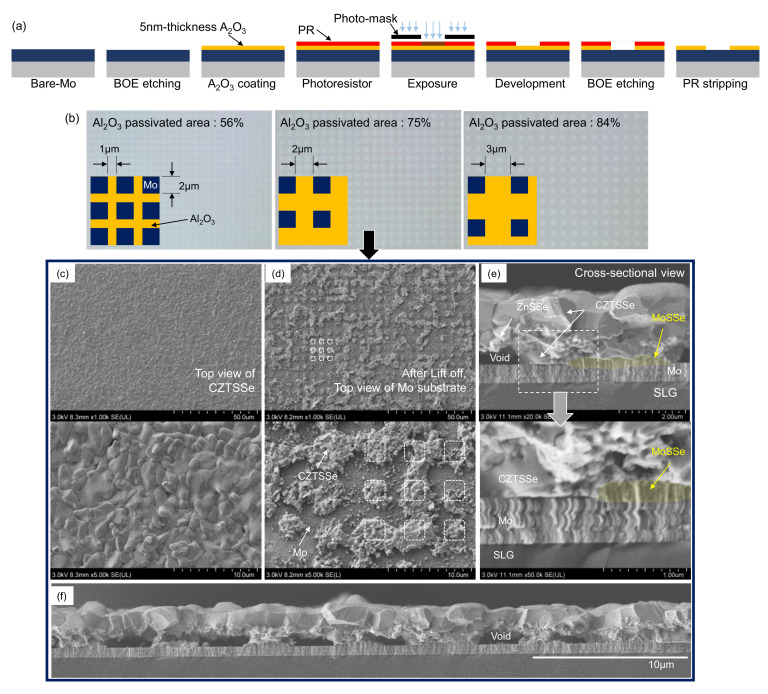
(**a**) Sequence of Al_2_O_3_ dot patterning by the photolithography process. (**b**) Photographs of the dot-patterned Al_2_O_3_/Mo substrate and its photo-mask pattern (Al_2_O_3_ passivated ratio is 56, 75, and 84%, respectively). FESEM images of (**c**) top view, (**d**) top view of Mo substrate after lifted-off, and (**e**) and (**f**) cross-sectional view of a sample with a passivation area of 75%.

Through applying the photolithogtaphy process, the effect of the 5-nm- Al_2_O_3_ dot patterning distribution on the CZTSSe solar cell efficiency was investigated, as shown in Figure 1a. Since MoOx forms easily, etching by the BOE solution was first conducted. As a result, the wetting angle (contact angle) of the droplet on the BOE-etched Mo surface ranged from 16.64° to 12.23°, as shown in Figure S1. And then, the 5-nm-thickness Al_2_O_3_ layer is deposited using ALD; the wetting angle of the droplet on the 5-nm-Al_2_O_3_ coated Mo surface is observed as 27.07°. Then, the general photolithography process is conducted to make the patterning in order as shown in Figure 1a. The following were conducted in order: Photoresistor coating, exposure using photo-mask, development for the patterning of the photoresistor, etching of the 5-nm-Al_2_O_3_ layer, and finally stripping the photoresistor. Photographs of the patterned 5-nm-Al_2_O_3_ coated Mo substrates are shown in Figure 1b with its photo-mask design. The patterning by photolithography is rough, owing to the poor resolution of the development process. However, the passivated-ratio trends can be expected by the photo-mask; the passivated-ratio of the photo-masks is 56, 75, and 84%, respectively.

Figure 1c–f show FESEM images of the CZTSSe samples using substrates with 75% 5-nm-Al_2_O_3_ passivated-area. FESEM images of the CZTSSe samples using substrates with 56% 5-nm-Al_2_O_3_ passivated-area are shown in Figure S2. Figure 1c shows the CZTSSe top view. Compared with samples without 5-nm-Al_2_O_3_ patterning, the relatively upper surface morphology appears to be flattened. A previous study found that when large voids were formed in the bottom CZTSSe layer, the grain size of the upper CZTSSe formed on the top was small, and the thickness was thin [33]. When large voids were not formed in the bottom CZTSSe layer, the grain size of the upper CZTSSe formed on the top was large and thick [33]. Hence, when Sn/Cu/Zn/Mo stacked precursor was used to synthesize the CZTSSe film, the fluctuating morphology of the CZTSSe film was formed. However, when the Sn/Cu/Zn/ Al_2_O_3_/Mo stacked precursor was used to synthesize the CZTSSe film, the flat morphology of the CZTSSe film was observed. These results may have been affected by the regular arrangement of voids. The CZTSSe layer was lifted off using epoxy to confirm the distribution of bottom CZTSSe; this method is shown in Figure S3. Figure 1d shows the Al_2_O_3_ patterned Mo surface microstructure remaining after exfoliation. CZTSSe layer delamination appears to occur at the CZTSSe/Al_2_O_3_ interface or the upper CZTSSe/bottom CZTSSe interface, as shown in Figure 1d. Figure 1e,f shows a cross-sectional FESEM image. MoSSe growth appears to occur only in the Mo-exposed area and seems to completely inhibit MoSSe growth in the region where 5-nm Al_2_O_3_ is passivated. Also, although the bottom CZTSSe is arranged in the Mo-exposed area, an area in which the bottom CZTSSe exists in the Al_2_O_3_ coated area is sometimes found. It was confirmed that the bottom CZTSSe is well connected to the Mo-exposed area in a dot pattern, and the growth of MoSSe was well suppressed through the 5-nm-Al_2_O_3_ layer.

**Figure 2:**
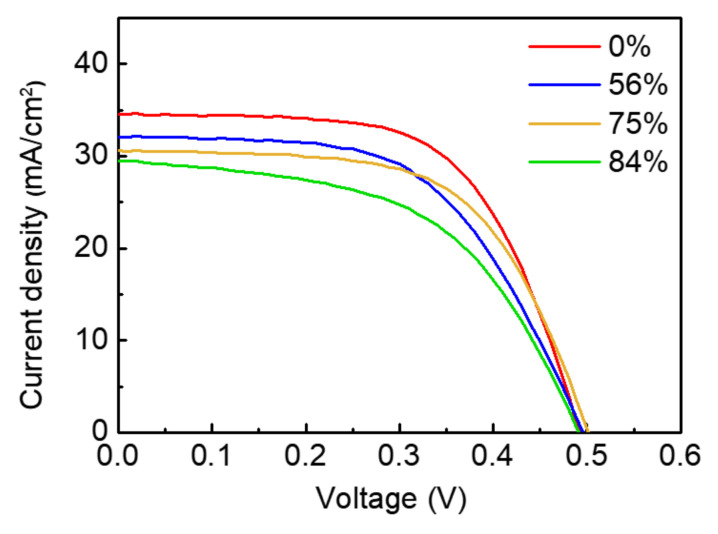
I–V curves of the best efficiency of the CZTSSe cell device, coated with different passivation ratios.

Using a series of 5-nm-Al_2_O_3_ coated Mo substrates, the CZTSSe solar cells were fabricated and characterized, as shown in Figure 2 and Table 1. It was observed that the efficiency decreases because Jsc decreases with the Al_2_O_3_ passivated area. FF tended to decrease slightly, and Voc changes according to the passivation area were insignificant. Due to the nature of our CZTS fabrication process, the absorber layer without patterning is expected to be in direct contact with Mo only about 50–60% of the CZTSSe lower part [15]; the remaining about 40–50% have voids unevenly distributed at the CZTSSe/Mo interface [15]. As shown in Table 1, the effect of increasing Voc due to the back-contact passivation seems to be insignificant (for reference, the dispersion range of Voc value within the same CZTSSe sample in our laboratory is about ~0.01 V). This may be because many voids still exist between the Al_2_O_3_ and the absorption layer, and thus the passivation by Al_2_O_3_ was not well performed. When the passivated area increased, the FF tend to decrease. It may be due to insufficient back contact formation. If all the 16% areas exposed to Mo serve as CZTS contacts, there will be no big problem (normally, in the case of CIGS, the passivation area is set up to the 95% level [34]). However, in the case of CZTS, since the Zn-related secondary phase may exist between upper CZTSSe and lower CZTSSe, it may interfere with the current flow. If the contact area is small, the degree of interference will be large. When the contact area initially provided is 16% (84% passivated device), it is estimated that the presence of the Zn-related secondary phase interferes with the role of the contact, resulting in a significant reduction in FF. The decrease in J_SC_ is likely to be due to changes in the electro-optical properties of the film such as average composition, local composition, crystallinity, thickness fluctuation, and surface morphology (reflectivity). The above characteristic changes are related with Na diffusion [35,36,37,38,39,40,41,42,43]. It was thought that sufficient Na diffusion did not occur through the Al_2_O_3_ intermediate layer. Thus, the distribution of Na composition in the CZTSSe layers was analyzed by TOF-SIMS.

**Figure 3:**
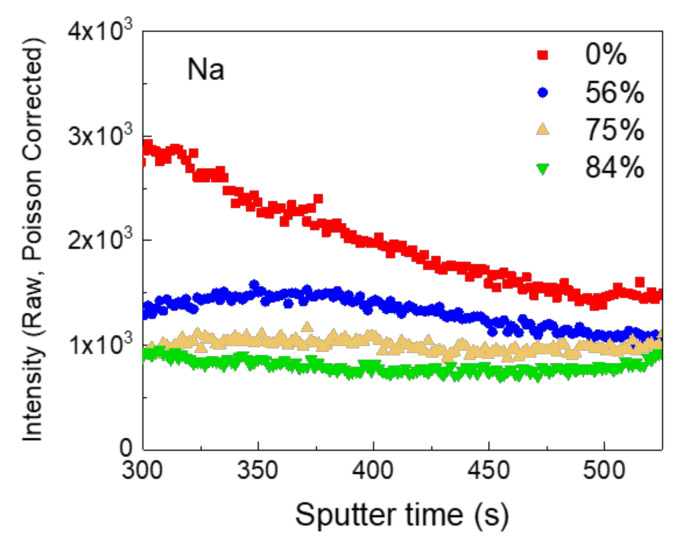
TOF-SIMS depth-profile of Na in the CZTSSe layer parts in the solar cell device, was coated with different passivation ratios.

As shown in Figure 3, the intensity of Na composition in the CZTSSe layer of the fully open cell is strongest, and the intensity of the Na decreases when the 5-nm-Al_2_O_3_ passivated area increases. The full TOF-SIMS spectrums of CZTSSe devices are found in Figure S4. So far, there have been reports about the effect of Na on the formation of high-quality CZTSSe. In CZTSSe, it has been reported that Na affects grain growth [35,36,37], defect passivation [38,39,40], increase in the p-type carrier concentration [41], prolonging the lifetime of the photo-generated carrier [42], and the reduction of non-radiative recombination [42,43]. However, the results of our TOF-SIMS analysis show that the amount of Na present in the CZTSSe absorber layer decreases with increasing Al_2_O_3_ passivated-area. In the results of CIGS solar cells, it was reported that when Na was not sufficiently diffused by Al_2_O_3_ patterning, FF and Voc decreased, and this tendency was improved by adding NaF [30,34,44]. In general, when using Al_2_O_3_ as an intermediate layer in the field of CIGS, it is known that Voc, Jsc, and FF increase for the following reasons. 1) The interface’s defect passivation effect is observed when the Al_2_O_3_ interlayer is applied to the CIGS/Mo interface [31,44]. 2) The passivated emitter and rear cell (PERC) effect applied to Si solar cells were expected by applying the Al_2_O_3_ layer to the CIGS/Mo interface in CIGS solar cells [30,31]. This negative charge reflection effect is expected to play an important role in addition to the band grading effect [31]. 3) Research has been conducted to collect the gain by the light reflectance effect in thin CIGS by inserting MgF_2_ into the intermediate layer together with Al_2_O_3_ [45]. Similarly, in CZTSSe solar cells, it has also been reported that the effect of interface defect passivation occurs when Al_2_O_3_ is applied to the void-free CZTSSe layer, increasing Jsc, Voc, and FF [32]. The following points should be considered to maximize the Al_2_O_3_ interlayer passivation effect. All of these Al_2_O_3_ interlayers need open points for the photo-generated holes to escape. Patterning for open points is known to require design based on the diffusion length, which derives the carrier lifetime in the light absorption layer. For example, in some cases, the pattern design of the back-contact passivation was decided based on the diffusion length of the minor carrier [34]. In these cases, the opening size was about a quarter of the diffusion length of the minor carrier, and the distance of the dot to the dot was designed to be twice as long as the diffusion length of the minor carrier [34]. The reason that the diameter and distance are given in a specific ratio is presumably to prevent minor carriers from acting as leakage currents. The carrier lifetime of photo-generated carriers obtained from TRPL analysis in CZTSSe is excitation-intensity-dependent, voltage-dependent, and temperature-dependent [46,47]. In addition, carrier trapping, surface effects, and energetic relaxation of carriers are generally involved in PL transitions, making it difficult to define carrier lifetime simply [46,47]. The diffusion length of CZTS derived from the photo-Hall effect is reported to be 0.75–1.5 μm [48]. Based on the reported diffusion length, the suitable dot diameter might be and the distance between dots might be 200–400 nm, and 1.5–3 μm respectively. Our patterning design has a small line width that is at the limit of the general aligner but, referring to the above, it is necessary to reduce both the dot size and distance between the dots further. To design such a pattern, it is required to use a stepper or e-beam lithography process, which is more advanced than the current equipment.

Our results that bottom CZTSSe of the CZTSSe double layer can be aligned by the patterning of the Al_2_O_3_ intermediate layer show the possibility of effectively solving the problems found in CZTSSe synthesized using conventional metal precursors; the problems are about locally compositional non-uniformity, non-uniform distribution of secondary phase, and non-uniform distribution of large voids. Also, the result that the Al_2_O_3_ passivation layer perfectly suppressed the formation of the MoSSe layer shows a possibility of efficiency improvement through the increase of the light reflection from the Mo electrode.

Here is how to solve some of the problems at this step. First, additional NaF should be applied to compensate for the Na deficiency. In this study, it was confirmed that the distribution of Na in the thin film was different depending on the Al_2_O_3_ passivation area. However, since Na directly affects grain growth and can provide CZTSSe having different bulk properties, it is difficult to simply explain the change of device characteristics due to the different Na content. Thus, to verify the role of additional NaF, it is necessary to separate the factor for Na among several factors in the future work. Second, the optimized pattern using a stepper or e-beam lithography should be designed to collect the photo-generated carriers effectively. If an efficient pattern design using the e-beam process is confirmed and commercialization needs to be considered, several other alternative processes can be used, such as PDMS stamping process (nano-imprinting).

In this study, the effect of 5-nm-Al_2_O_3_ dot patterning on the CZTSSe solar cell efficiency was investigated. It was confirmed that the 5-nm Al_2_O_3_ pattern could completely inhibit MoSSe generation, and the bottom CZTSSe distribution was well controlled by dot patterning of the 5-nm-Al_2_O_3_ layer. However, the efficiency, Jsc, Voc, and FF tended to decrease when the Al_2_O_3_ passivated area increased; the origin of the decrease in cell characteristics was expected to be inadequate Na diffusion from SLG and the insufficient optimized pattern. Nevertheless, it was found that void self-arrangement occurs, which is of great significance in that it will be an important method to improve the local compositional non-uniformity that occurs when there is no pattern. Also, in the future work, it is necessary to apply an effective local contact structure to obtain the optical J_SC_ gain at the rear of the CZTS. When Al_2_O_3_ is used, MoSe_2_ formation is prevented, and thus J_SC_ gain can be additionally expected through increased reflection from the rear surface. In addition, in order to maximize the optical reflection effect at rear surface, a new passivation layer having excellent reflection property can be applied. The structure of the solar cell applying new passivation layer that maximizes the reflection effect while the voids are arranged on the passivation layer is a new structure that has never been. This unique solar cell structure may help break the current CZTS recording efficiency.

## Figures and Tables

**Table 1:** Open circuit voltage (Voc), short-circuit current density (Jsc), Fill Factor (FF), and power conversion efficiency (Eff) of the CZTSSe devices fabricated with different closed-ratios of Al_2_O_3_ passivated areas.

Al_2_O_3_ Passivated Area (%)	V_OC_ [V]	J_SC_ [mA/cm^2^]	FF [%]	Eff [%]
0	0.493	34.58	61.22	10.43
56	0.496	32.09	56.16	8.94
75	0.502	30.61	60.37	9.27
84	0.492	29.54	52.82	7.67

## Supplementary Materials

The following are available online at https://www.mdpi.com/2079-4991/10/9/1874/s1, Figure S1: Wettability of bare-Mo, BOE-etched Mo, and Al_2_O_3_ coated Mo; Figure S2: FESEM images of (a) top view and (b) bottom view of lifted-off CZTSSe of the sample with a passivation area of 56%; Figure S3: Lift-off process of CZTSSe cell, and Figure S4; Depth-profiles of each CZTSSe device by TOF-SIMS.
