# Supplementary material for: Effect of Al2O3 Dot Patterning on CZTSSe Solar Cell Characteristics
Source: Nanomaterials (Basel). 2020 Sep 18;10(9):1874. doi: 10.3390/nano10091874 (PMC7557866; doi:10.3390/nano10091874)
Supplement: Supplementary file 1 [file nanomaterials-10-01874-s001.pdf]

nanomaterials

Supporting information

Effect of Al_2O_3 Dot Patterning on CZTSSe Solar Cell Characteristics

Se-Yun Kim [1,2], Sanghun Hong [3], Seung-Hyun Kim [1], Dae-Ho Son [1,4], Young-Ill Kim [1], Sammi Kim [1], Young-Woo Heo [3], Jin-Kyu Kang [1,4,*] and Dae-Hwan Kim [1,4,*]

[1] Research Center for Thin Film Solar Cells, Daegu-Gyeongbuk Institute of Science and Technology (DGIST), Daegu 42988, Republic of Korea; kimseyun@kyungnam.ac.kr (S.-Y.K.); seunghyun@dgist.ac.kr (S.-H.K.); dhson@dgist.ac.kr (D.-H.S.); lynx012@dgist.ac.kr (Y.-I.K.); smkim@dgist.ac.kr (S.K.)

[2] Department of Nano Materials Science and Engineering, Kyungnam University, Gyeongsangnam-do 51767, Republic of Korea

[3] School of Materials Science and Engineering, Kyungpook National University, Daegu, 41566, Republic of Korea; shhong@dgist.ac.kr (S.H.); ywheo@knu.ac.kr (Y.-W.H.)

[4] Division of Energy Technology, Daegu-Gyeongbuk Institute of Science and Technology (DGIST), Daegu 42988, Republic of Korea

* Correspondence: apollon@dgist.ac.kr (J.-K.K.); monolith@dgist.ac.kr (D.-H.K.)

Keywords: CZTSSe; metal precursor; two-step process; back-contact passivation; void arrangement

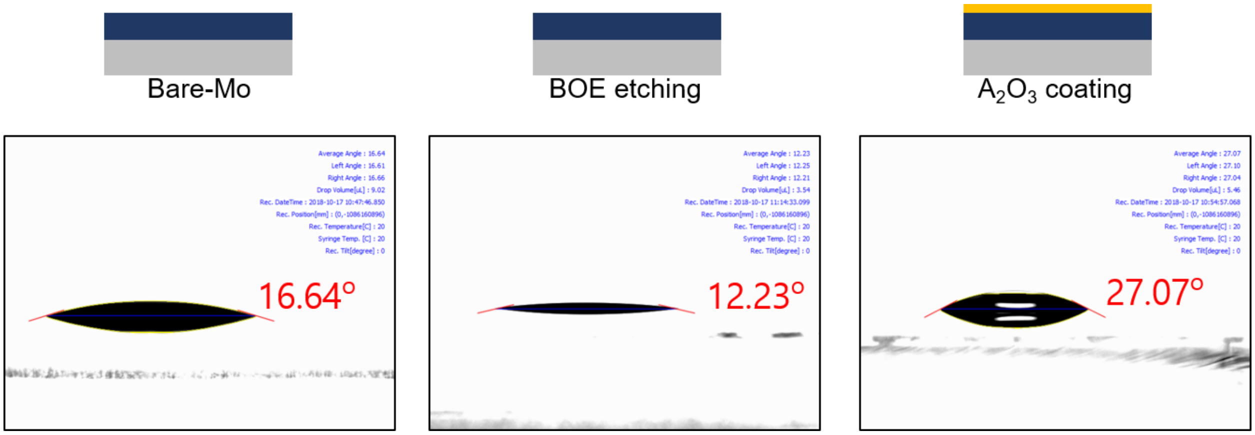

Figure S1. Wettability of bare-Mo, BOE-etched Mo, and Al_2O_3 coated Mo.

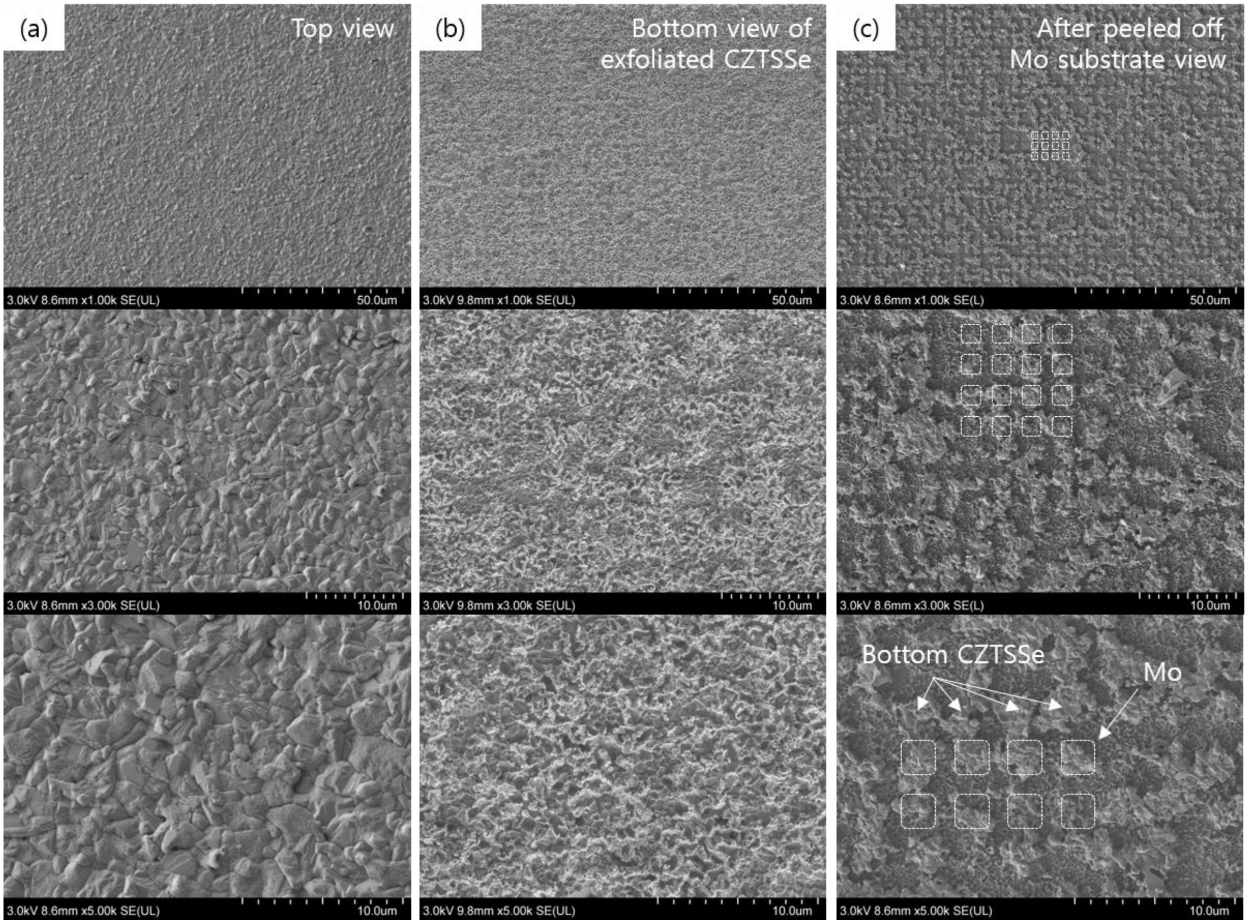

Figure S2. FESEM images of (**a**) top view, (**b**) bottom view of lifted-off CZTSSe of the sample with a passivation area of 56%. (**c**) Top view of the Mo substrate after lifted-off.

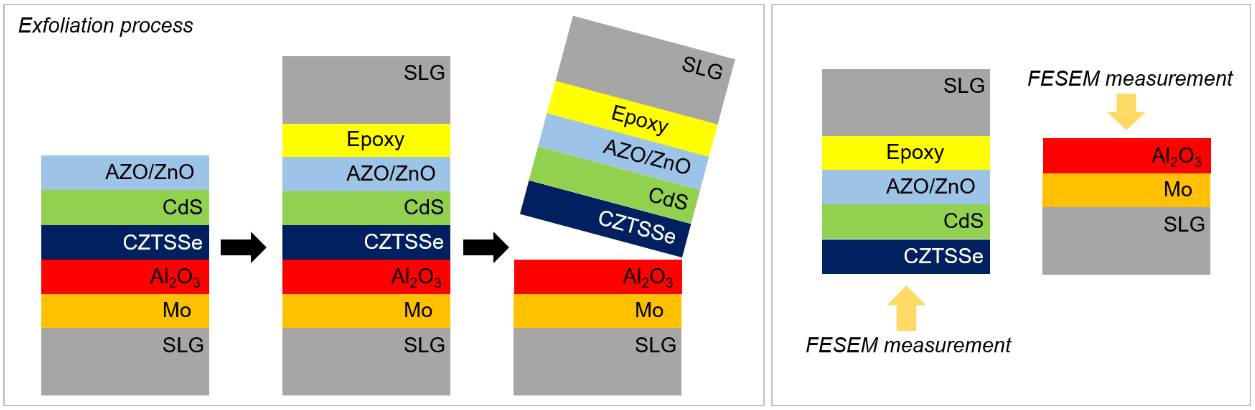

Figure S3. Lift-off process of CZTSSe cell.

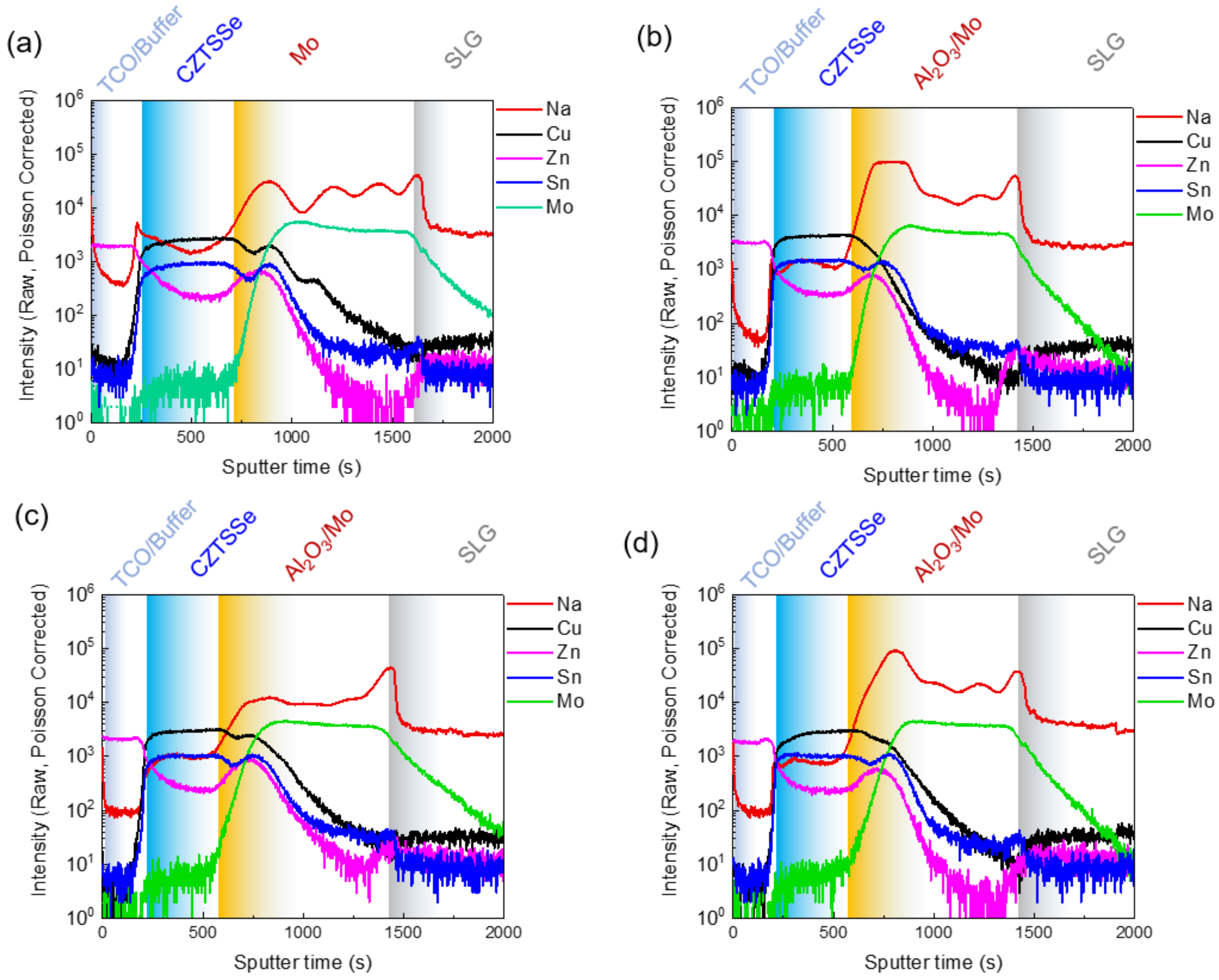

Figure S4. Depth-profiles of each CZTSSe device by TOF-SIMS. The passivation ratio (**a**)0, (**b**)56, (**c**)75, and (**d**)85 %.
